# Detection of oral *Helicobacter Pylori* infection using saliva test cassette

**DOI:** 10.12669/pjms.315.7626

**Published:** 2015

**Authors:** Min Yu, Xue-Yan Zhang, Qing Yu

**Affiliations:** 1 Min Yu, MD. Yancheng Institute of Health Sciences, Yancheng, Jiangsu 224005, China; 2 Xue-Yan Zhang, MD. Yancheng Institute of Health Sciences, Yancheng, Jiangsu 224005, China; 3 Qing Yu, MD. Yancheng Institute of Health Sciences, Yancheng, Jiangsu 224005, China

**Keywords:** *Helicobacter pylori*, Saliva Test Cassette, Oral risk factors. Epidemiology

## Abstract

**Objective:**

To investigate the incidence of oral infection with *Helicobacter pylori* (*H. pylori*) and identify related epidemiological factors among freshmen of four colleges in Yancheng.

**Methods:**

The data, scored positive or negative, were collected on 160 individuals who had been diagnosed by *H. pylori* Saliva Test Cassette (HPS) during October 2013 to October 2014. *H. pylori* Saliva Test Cassette (HPS) is to use colloidal gold technique to specifically identify urease in saliva. A standard questionnaire, with variables including sex, educational degree of parents etc., was used in the subjects. Statistical data of diagnostic test were analyzed by SPSS17.0 software.

**Results:**

Out of 160, 82 subjects were detected positive and 78 were negative. In univariate analysis, dental plaque, family history of stomach diseases, habit of washing hands before meals and habit of brushing teeth twice daily were associated negatively with *H. pylori* infection. Multivariate logistic regression analysis showed that dental plaque and family history of stomach diseases were the risk factors which may be associated with *H. pylori* infection.

**Conclusions:**

Dental plaque and family history of gastric diseases were risk factors of oral *H. pylori* infection. It is vital for the prevention of *H. pylori* infection to focus on health education and oral hygiene, and avoid transmission by oral-oral route as well.

## INTRODUCTION

It has attracted extensive attention that *Helicobacter pylori* infection correlates closely to chronic gastritis, peptic ulcer and gastric cancer.^1^,^2^ *H. pylori* were considered as group 1 carcinogen by the International Agency for Research on Cancer (IARC) in 1994. Recently, researchers have discovered that there may be some relativity between *H. pylori* infection and many extragastro intestinal diseases, such as hematological, skin, and cardiovascular and respiratory system diseases.^3^-^6^

Diagnostic tests of *H. pylori* infection include invasive and noninvasive methods. Noninvasive tests such as immunoassay for serological antibodies against *H. pylori*, ^13^C Urea Breath Test and ^14^C-Urea Breath Test (UBT), are more acceptable. Traditional serological test mainly detects the specific IgG antibody against *H. pylori* in serum, but can’t distinguish current infection from previous infection.^7^ UBT mainly identifies *H. pylori* infection in gastric mucosa by determining the amount of ^13^CO_2_ or ^14^CO_2_ in breath, by means of measuring equipment with high sensitivity after subjects intake a certain amount of ^13^C or ^14^C–labeled urea.^7^-^8^ Saliva *H. pylori* antibody testing and stool antigen testing can readily be manipulated, as many studies have reported.^9^,^10^ However, it was seldom reported regarding saliva *H. pylori* antigen testing.^11^ *H. pylori* Saliva Test Cassette (HPS) is to use monoclonal antibody to specifically identify urease in saliva after *H. pylori* infection. Its sensitivity is 10ng/mL. Although many bacteria in oral cavity can produce urease, common oral bacteria were analyzed and did not show interference or cross-reactivity with the test. A number of researches have compared HPS to UBT/RUT,^12^,^13^ and shown that there is a correlation between results of ^13^C-UBT and HPS. Combination of ^13^C-UBT and HPS may compensate for the blind zone of ^13^C-UBT detecting oral *H. pylori* infection. ^13^C-UBT should not be a gold standard to judge the validation of HPS in detecting oral *H. pylori* infection. HPS is a new noninvasive method, which is especially applicable to detect oral *H. pylori* infection.

In order to investigate the state of oral infection with *H. pylori* and identify the correlation between *H. pylori* infection and epidemiological factors (*e.g*., eating habits, oral hygiene and disease history, etc.) among students of four colleges in Yancheng, we utilized *H. pylori* Saliva Test Cassette to test 160 newly admitted patients and administrated a questionnaire. We expect to improve the knowledge of *H. pylori* infection among youngsters, help those with high risk factors attach importance to periodic checks, and provide basis for prevention programs as well.

## METHODS

Subjects were provided with a written information letter about the study who gave their informed consent. Ethics committee of Yancheng Institute of Health Sciences approved our study design, methods and the consent procedure used for this study which was conducted during October 2013 to October 2014.

### Subjects

Using stratified random sampling method, we randomly selected 160 fresh students (69 male and 91 female, mean age 18.38±0.85) enrolled in 2013 from the four colleges in Yancheng as respondents. They were free from medication with PPIs or histaminereceptor antagonists for a minimum of 2 weeks and antibiotics or colloidal bismuth subcitrates for 4 weeks prior to testing.^7^

### Detection method

The *H. pylori* Saliva Test Cassette (Ameritek Diagnostic Reagent Co., Ltd, Jiaxing, China) was used to detect the oral infection of *H. pylori*. The subjects were not allowed to brush their teeth, rinse their mouths, drink water or take foods one hour prior to the test. A minimum of 0.5 mL of saliva was collected into a disposal testing cup each and the test was performed within 5 minutes. According to the manual, we added the mixture of saliva and buffer solution into the sample well, and observed the result within 20-30 min. As the test kit began to work, red color was seen to move across the result window in the center of the test disk. The presence of 2 red color bands (‘T’ band and ‘C’ band, i.e., testing band and control band) within the result window indicated a positive result. The presence of only 1 color band indicated a negative result. If there was no control band, the sample should be retested.^13^

### Investigation method

A standard questionnaire was administrated to the subjects. Characteristics include sex, educational degree of parents (university or less than university degree), frequency of eating out weekly (≥4 or <3), consumption of fresh vegetables and fruits (daily or not daily), habit of hand washing before meals (frequent or seldom), brushing habit (twice daily or once daily), dental plaque, dental caries and family history of stomach diseases. The diagnoses of dental plaque and dental caries were established on the basis of entrance physical examination. Family history of stomach diseases included chronic gastritis, peptic ulcer and gastric cancer diagnosed by clinical practice doctors. All the 160 subjects signed informed consents and completed the investigation.

### Statistical analysis

Data analysis was conducted using SPSS 17.0 software. A univariate analysis was conducted to explore how each characteristic is associated with *H. pylori* infection. Statistical significance was determined using Chi-square tests and was defined as a *p*-value of <0.05. We included variables that were significant in univariate analysis to conduct a multivariate logistic regression analysis. The regression model was using “Enter” method. The inclusion criteria were 0.05 and the exclusion criteria were 0.10.

## RESULTS

A total of 82 subjects were detected positive and 78 were negative. The oral *H. pylori* infection rate among them was 51.25%. Original data were scored positive and negative. Other data are all listed in Table-I and II.

**Table-I:** Univariate analysis of risk factors for oral *H.pylori* infection.

Variables	H.pylori		χ^2^	p	OR	95% CI
	+	-				
Sex			0.041	0.839	1.067	0.571-1.996
Male	36	33
Female	46	45
Education degree of parents			1.511	0.219	1.510	0.781-2.920
Less than university degree	58	48		
University	24	30		
Frequency of eating out weekly			2.427	0.119	1.645	0.878-3.081
≥4	50	38
<3	32	40
Intake of fresh vegetables and fruits			0.982	0.322	1.407	0.715-2.770
Daily	28	21			
Not daily	54	57			
Habit of washing hands before meals			4.838	0.028	0.486	0.254-0.928
Frequent	25	37				
Seldom	57	41				
Habit of brushing teeth			4.722	0.030	0.490	0.257-0.936
Twice daily	43	54				
Once daily	39	24				
Dental plaque			5.425	0.020	2.245	1.129-4.463
Yes	33	18				
No	49	60				
Dental caries			0.722	0.396	0.658	0.250-1.735
Yes	8	11				
No	74	67				
Family history of stomach diseases			5.983	0.014	2.811	1.202-6.572
Yes	22	9				
No	60	69				

**Table-II:** Multivariate logistic regression analysis of risk factors for oral *H.pylori* infection.

Variables	Wald	p	OR	95% CI
Washing hands before meals	1.502	0.305	0.684	0.331-1.413
Brushing teeth twice daily	3.214	0.073	0.509	0.244-1.605
Dental plaque	4.617	0.032	2.195	1.072-4.498
Family history of stomach diseases	4.063	0.044	2.456	1.025-5.886

### Univariate analysis

All variables were analyzed by univariate using Chi-square tests. At level of α=0.05, risk factors associated with oral *H. pylori* infection were preliminarily screened: dental plaque (OR=2.245, 95% CI: 1.129-4.463, *p*=0.020), and family history of stomach diseases (OR=2.811, 95% CI: 1.202-6.572, *p=*0.014). Protective factors included: habit of washing hands before meals (OR=0.486, 95% CI: 0.254-0.928, *p*=0.028), and habit of brushing teeth twice daily (OR=0.490, 95% CI: 0.257-0.936, *p*=0.030). The following factors were not significantly associated with oral *H. pylori* infection: sex, parents’ education less than university degree, frequency of eating out weekly, not intaking vegetables and fruits daily, and dental caries (Table-I).

### Multivariate analysis

Multivariate logistic regression analysis model included: habit of washing hands before meals, brushing teeth twice daily, dental plaque, and family history of stomach diseases. Among which, habit of washing hands before meals, and brushing teeth twice daily were removed from regression equation. Dental plaque (OR=2.195, 95% CI: 1.072-4.498, *p*=0.032), and family history of stomach diseases (OR=2.456, 95% CI: 1.025-5.886, *p*=0.044) were proved as independent risk factors of oral *H.pylori* infection (Table-II).

## DISCUSSION

We used *H. pylori* Saliva Test Cassette (HPS) for epidemiological investigation in this study. We sought to choose an economical and noninvasive method to test the *H. pylori* infection among our students. At first, serological tests and stool tests were taken into account. But we excluded serological tests due to its less accuracy.^7^ As regards stool antigen tests, we were worried about the decline of sensitivity with samples left at room temperature.^14^ The method of UBT has always been considered as the gold standard to judge *H. pylori* infection, to judge *H. pylori* infection in stomach, based on its principle. The method of HPS has the advantage of being economical and simplicity, moreover, its specificity and sensitivity in detecting *H. pylori* in oral cavity had been confirmed.^13^ Positive results of HPS can indicate the occurrence of oral infection alone or co-infection of gastric and oral *H. pylori*.

In previous publications regarding the detection of *H. pylori* infection in dental plaque and saliva, clinical patients were recruited as study population^15^,^16^ and there were no references pertaining to the oral infection status in the general population. The oral *H. pylori* infection rate among our participants was 51.25%.

Our study didn’t find that regular consumption of fresh fruits and vegetables can reduce the risk of oral *H. pylori* infection, although several studies have suggested high intake of fresh fruits and vegetables may reduce the risk of gastric cancer.^17^,^18^ As Herrera thought, a risk of transmitting *H. pylori* to humans may be caused by intake of contaminated vegetables.^19^ Our study also showed that good hygiene habits, like washing hands before meals and brushing teeth twice daily, played a role in the prevention of oral *H. pylori* infection.

In the present study, dental plaque and family history of gastric diseases were risk factors of oral *H. pylori* infection, which suggested that oral diseases may have correlation with *H. pylori* infection and there existed a familial clustering phenomenon of *H. pylori* infection. Our findings extend the evidence from previous reports that oral-oral transmission may be a primary route of *H. pylori* infection.^20^

To minimize the risk of *H. pylori* infection, it is recommended to change poor health habits and pay attention to dietary hygiene and oral hygiene. What’s more important is to see the dentist to remove dental plaque and calculus in time, and to separately eat by dishes to avoid cross infection by fecal-oral or oral-oral transmission among family members.^21^ Our students may also strengthen health education in their families and communities. Since the possibility of infection developing into peptic ulcer and gastric cancer will continue to be a critical problem in the future,^22^ those who have gastrointestinal symptoms with positive HPS results require further testing of gastric *H. pylori* infection. If there are signs of *H. pylori* infection in stomach, treatment is needed. Moreover, since *H. pylori* colonized in oral cavity might be one of the major causes of the recurrence after eradication therapy,^23^ it is worthwhile to note that we should eradicate *H. pylori* in oral cavity at the same time when preventing recurrence.

## CONCLUSIONS

*H. pylori* Saliva Test Cassette was used to detect the oral *H. pylori* infection rate and results showed that dental plaque and family history of gastric diseases were risk factors of oral *H. pylori* infection. Taking the possibility of inducing gastric infection into account, we need to establish and keep good hygiene habits, especially oral hygiene, and avoid cross infection among family members.
